# Clinical Features and Prevalence of Spondyloarthritis in a Cohort of Italian Patients Presenting with Acute Nongranulomatous Anterior Uveitis

**DOI:** 10.1155/2022/6632081

**Published:** 2022-01-18

**Authors:** Elena Bolletta, Pierluigi Macchioni, Giorgia Citriniti, Valentina Mastrofilippo, Raffaella Aldigeri, Luca De Simone, Fabrizio Gozzi, Chantal Adani, Anna Sangiovanni, Chiara Posarelli, Michele Figus, Francesco Muratore, Nicolò Pipitone, Carlo Salvarani, Luca Cimino

**Affiliations:** ^1^Ocular Immunology Unit, Azienda USL-IRCCS of Reggio Emilia, Reggio Emilia, Italy; ^2^Rheumatology Unit, Azienda USL-IRCCS of Reggio Emilia, Reggio Emilia, Italy; ^3^Department of Medicine and Surgery, University of Parma, Parma, Italy; ^4^Department of Surgical, Medical and Molecular Pathology and Critical Care Medicine, University of Pisa, Pisa, Italy; ^5^Department of Surgery, Medicine, Dentistry and Morphological Sciences, with Interest in Transplants, Oncology and Regenerative Medicine, University of Modena and Reggio Emilia, Modena, Italy

## Abstract

**Purpose:**

To describe the clinical features of acute nongranulomatous anterior uveitis (NGAU) patients and to estimate the prevalence of concomitant spondyloarthritis (SpA).

**Methods:**

Retrospective study of consecutive patients affected by NGAU referred to the Ocular Immunology Unit of the AUSL-IRCCS di Reggio Emilia, Italy, between January 2016 and January 2019. All patients underwent ophthalmic evaluation and blood test with HLA-B27 typing and were referred to a rheumatologist to identify any undiagnosed SpA. SpA was classified according to the Assessment of SpondyloArthritis international Society (ASAS) criteria in axial or peripheral SpA. Patients were divided into two groups: NGAU with associated SpA (SpA+) and NGAU without SpA (SpA-). Clinical and demographic features of the two groups, including sex, HLA-B27, family history of rheumatic disease, uveitis laterality, course, and severity of ocular inflammation, complications, and treatment, were compared.

**Results:**

Ninety-nine patients with NGAU were enrolled, of whom 36 (36%) with a diagnosis of SpA: 14 with peripheral SpA and 22 with axial SpA. The prevalence of SpA was higher in HLA-B27-positive patients than in HLA-B27-negative patients (50% vs. 15%, *p* < 0.0001). The multivariate logistic regression (*R*^2^ = 0.28) for SpA diagnosis identified as significant predictive factors: age at diagnosis (odds ratio [OR] = 0.95, 95% confidence interval [CI]: 0.91-0.99) and HLA-B27+ (OR = 5.32, 95% CI: 1.80-15.70).

**Conclusions:**

Our results confirmed the high prevalence of undiagnosed SpA in patients with NGAU, suggesting that, regardless of HLA-B27 status, in the presence of IBP and/or peripheral arthritis, patients with NGAU must be referred to the rheumatologist to allow earlier diagnosis.

## 1. Introduction

Acute anterior uveitis (AAU) is the most common type of uveitis worldwide, with higher prevalence in Western countries, where it accounts for approximately 50-92% of cases [1]. According to recent epidemiological studies, it represents 49-58% of uveitis in Italy [2–4].

A distinction between granulomatous and nongranulomatous uveitis is mandatory for a correct diagnostic evaluation of anterior uveitis [5]. Most cases of acute nongranulomatous anterior uveitis (NGAU) are associated with autoimmune diseases, primarily spondyloarthritis (SpA) [6]. AAU in SpA patients typically occurs as recurrent or alternating unilateral NGAU with conjunctival and ciliary injection, resulting in the visible redness of the affected eye. A clinical assessment of intraocular inflammation by slit-lamp examination shows intraocular cells and the presence of protein exudation in the aqueous humor of the anterior chamber. This can lead to direct leukocyte sedimentation in the anterior chamber (“hypopyon”) and to the formation of posterior synechiae as a secondary sequela, resulting in adhesions between the iris and the lens [7].

SpA is also known as seronegative spondyloarthropathies, a family of rheumatic diseases that include ankylosing spondylitis, psoriatic arthritis, arthritis associated with inflammatory bowel disease (IBD), reactive arthritis (formerly known as Reiter's syndrome), and undifferentiated SpA [8].

AAU prevalence in SpA ranges from 21% to 33%, according to a recent meta-analysis [7]. Other studies report the prevalence of previously undiagnosed SpA in AAU ranging from 40% to 67%; this percentage is even higher when uveitis is associated with the human leukocyte antigen- (HLA-) B27 [9, 10].

Since AAU is the most frequent extra-articular manifestation of SpA and can be its first sign, ophthalmologists should refer these patients to a rheumatologist, although there are no shared guidelines defining which patients should be referred. The Dublin Uveitis Evaluation Tool (DUET) algorithm has been proposed to guide the selection of appropriate candidates [9]. Moreover, a Delphi consensus was undertaken to standardize red flags for referral to rheumatologists and to ophthalmologists of patients with rheumatic diseases and ocular involvement [11].

This study evaluated the ophthalmological and rheumatological clinical features as well as the demographics and genetics (HLA-B27) of patients with NGAU to differentiate between NGAU associated with SpA (SpA+) and NGAU without SpA (SpA-) and to help ophthalmologists to appropriately select patients for rheumatology referral.

## 2. Materials and Methods

Consecutive adult patients with NGAU referred to the Ocular Immunology Unit of the AUSL-IRCCS di Reggio Emilia, Italy, between January 2016 and January 2019 were enrolled in this retrospective study. Patients were referred by ophthalmologists, general practitioners, or rheumatologists.

NGAU is defined as acute onset uveitis with anterior chamber reaction without granulomatous keratic precipitates by the Standardization of Uveitis Nomenclature (SUN) Working Group guidelines [12]. Exclusion criteria in this study were the presence of other causes of uveitis, previously diagnosed SpA and/or no HLA test result available.

All patients underwent a complete ophthalmic evaluation, including best corrected visual acuity (BCVA), intraocular pressure (IOP), slit-lamp examination of the anterior segment with clinical evaluation of anterior chamber inflammation, and of the fundus after pupillary dilatation.

Blood test with HLA-B27 typing was also performed in all patients, and subsequently, they were referred to a rheumatologist to identify any undiagnosed SpA. A careful family history was taken to highlight any immune-mediated systemic diseases, including SpA, psoriasis, and IBD, i.e., Crohn's disease and ulcerative colitis (UC). Other parameters recorded were body mass index (BMI), smoking status, comorbidities, age at first episode of uveitis, age at diagnosis of SpA, and the presence and age at onset of inflammatory back pain (IBP), as defined by the Assessment of SpondyloArthritis international Society (ASAS) criteria. According to these criteria, IBP is chronic back pain (CBP) lasting more than 3 months, with four out of five of the following parameters: age at onset <40 years, insidious onset, improvement with exercise, no improvement with rest, and pain at night (with improvement upon getting up). SpA was classified according to the ASAS criteria as axial or peripheral SpA [13, 14]. Patients were divided into two groups: NGAU with associated SpA (SpA+) and NGAU without SpA (SpA-). Rheumatological examination included the Bath Ankylosing Spondylitis Disease Activity Index (BASDAI) to evaluate disease activity of axial SpA. Sacroiliac radiography or magnetic resonance imaging (MRI) and ultrasound of the entheses and peripheral joints were performed when requested by the rheumatologist.

The study was conducted according to the Declaration of Helsinki and was approved by the local Ethics Committee (2019/0100242). Informed consent was obtained from all patients at enrollment.

Statistical analysis was performed using the SPSS software, v.27. Significance was defined as *p* < 0.05 (two-tailed). To describe the study sample, we used frequencies, mean and standard deviation, or median and interquartile range, depending on the distribution. Comparisons were performed using *t*-test or chi-square test. The association between clinical variables and SpA was determined using Pearson's correlation coefficient and multivariate logistic regression model.

## 3. Results

Ninety-nine consecutive patients with NGAU were enrolled in the study: 38 males (38%) and 61 females (62%), with a mean age of 46 ± 13 years (range 20-75 years). Sixty (60%) subjects with NGAU were HLA-B27+. Most of the patients (71%) had recurrent uveitis, 40% presented alternant bilateral involvement, and 29% had posterior synechiae, the most frequent complication. The main characteristics of the patients and clinical findings are summarized in Table 1 and divided by sex in Tables 2 and 3.

A total of 36 (36%) patients were diagnosed with SpA: 14 patients with peripheral SpA and 22 with axial SpA (ASAS criteria) (Table 4).

No differences in BMI, sex, or smoking status were found between the SpA+ and SpA- groups.

Age at diagnosis of uveitis was significantly lower in SpA+ patients (*p* = 0.005). IBP and peripheral arthritis incidence were significantly higher in the SpA+ group (*p* < 0.001), as was HLA-B27 positivity (*p* < 0.001) (Table 1). No significant differences in ophthalmologic features, including recurrence of uveitis, laterality, hypopyon, synechiae, and cataract, were observed between SpA+ and SpA- patients.

Age at diagnosis of uveitis in patients with positive HLA-B27 was lower than in patients with negative HLA-B27 (mean age 40 vs. 45 years; *p* = 0.049^∗^) (Table 5).

The prevalence of both axial and peripheral SpA was higher in HLA-B27+ patients (19/60 [31.7%] and 11/60 [18.3%], respectively) than in HLA-B27- patients (3/39 [7.7%] and 3/39 [7.7%]) (*p* = 0.005∗∗ and *p* = 0.14) (Table 5).

The multivariate logistic regression (*R*^2^ = 0.28) for SpA diagnosis, which included age at diagnosis, sex, ocular bilateral involvement, acute/recurrent trend, and HLA-B27 positivity, identified as significant predictive factors of SpA age at diagnosis (OR = 0.95, 95% CI: 0.91-0.99) and HLA-B27+ (OR = 5.32, 95% CI: 1.80-15.70).

Twenty-eight subjects were previously treated with systemic steroids, while 92 with topical steroids (100% in SpA + vs. 88% in SpA-, *p* = 0.017). Eight patients were prescribed with biologics, 13% in the SpA+ group vs. 4% in the SpA- (*p* = 0.09). One SpA- patient was treated with biologic therapy with adalimumab for UC.

## 4. Discussion

Anterior uveitis is the most common extra-articular manifestation of SpA and is characterized by nongranulomatous clinical features. A well-structured screening of patients affected by NGAU could allow early diagnosis, considering that the mean diagnostic delay of SpA from symptom onset ranges from 4.9 to 6.8 years [15, 16]. It must be taken into account that delayed diagnosis is associated with worse spinal functional impairment, greater radiographic progression, reduced response to treatment, and poorer quality of life [17, 18].

The SENTINEL Interdisciplinary Collaborative Project was a multicenter prospective observational study on the largest cohort of patients with anterior uveitis who were systematically screened for associated and underdiagnosed SpA [9]. This and other previous studies report the high prevalence of undiagnosed SpA in patients with anterior uveitis: 67.7% by Juanola et al. and 40% by Haroon et al. [9, 10].

Our study confirms this high prevalence (36%), despite some differences in the inclusion criteria. The SENTINEL study excluded HLA-B27-negative patients with a single episode of AAU, while in our cohort, 11.1% of patients with newly diagnosed SpA met these exclusion criteria [9].

In accordance with the two above-mentioned studies, the prevalence of SpA was higher in our HLA-B27-positive patients than in those HLA-B27-negative (50% vs. 15%, *p* < 0.0001).

Moreover, the age at diagnosis of uveitis in HLA-B27-positive patients was significantly lower than in patients with negative HLA-B27, in accordance with the SENTINEL study.

The multivariate logistic regression analysis for SpA diagnosis underlined as significant predictive factors age at diagnosis and HLA-B27 positivity.

Similarly to the SENTINEL study, we did not detect any important differences in the ophthalmologic features between subgroups, contrary to the study by Power et al. [9, 19]. According to Zaidi et al., patients with both a spondyloarthropathy and HLA-B27 positivity tend to have a higher risk of hypopyon than either factor alone [20]. In our study, both patients with hypopyon were HLA-B27 positive and classified as having axial SpA.

Since there are no commonly accepted clinical practice guidelines for rheumatology referral, HLA-B27 positivity in patients with anterior uveitis often represents an indication to recommend a rheumatological evaluation [21, 22]. Haroon et al. proposed the DUET algorithm for ophthalmologists evaluating patients with AAU as a tool for rheumatology referral (DUET algorithm, sensitivity 95%, and specificity 98% [10]). According to this algorithm, the criteria suggesting rheumatologist referral are back pain lasting more than 3 months and onset at 45 years of age or older, or joint pain requiring a general practitioner's evaluation together with HLA-B27 positivity or coexisting psoriasis [10]. As this algorithm does not require any imaging evaluation such as sacroiliac X-rays or MRI and/or ultrasound of entheses and peripheral joints, ophthalmologists can easily use it in their daily clinical practice. In our cohort, 6 patients (17%) with newly diagnosed SpA had a history of IBP or joint stiffness and pain but were negative for HLA-B27 and psoriasis. By applying the DUET algorithm, these patients would not have been evaluated for SpA. Therefore, SpA must be taken into consideration in HLA-B27-negative patients with anterior uveitis as well.

This was further confirmed that, regardless of HLA status, associated undiagnosed SpA must be suspected in the presence of IBP and/or signs and symptoms of peripheral arthritis in patients with NGAU.

According to Olivieri et al., the screening of patients presenting with NGAU for rheumatology referral plays a key role in the diagnosis and management of SpA [11], and red flags for referral have been outlined. Patients suffering from acute anterior nongranulomatous noninfectious uveitis should be evaluated by a rheumatologist when chronic low back pain has lasted more than 3 months or when there is a family/personal history of psoriasis involving the skin and/or nails and/or of SpA and/or IBD and/or Behçet's disease and/or oral and/or genital aphthae or erythema nodosum [11].

Furthermore, Sykes et al., using MRI to search for the presence of axial SpA in unselected patients presenting with AAU, found a high prevalence of undiagnosed axial SpA in these patients, nearly half of whom were HLA-B27 negative. These authors concluded that there does not appear to be a simple mechanism for screening these patients, and given the significant burden of ‘hidden' axial SpA, recommend that ophthalmologists refer all patients with AAU with CBP onset at age ≤ 45 years to rheumatology for further evaluation regardless of HLA-B27 status, sex, or number of episodes of AAU [23].

Our results confirm the high prevalence of undiagnosed SpA in patients with NGAU, suggesting that close collaboration between ophthalmologists and rheumatologists could improve the management of these patients.

In conclusion, regardless of HLA-B27 status, in the presence of IBP, as defined by ASAS experts and/or because of signs and symptoms of peripheral arthritis, patients with NGAU must be referred to the rheumatologist to allow earlier diagnosis. The Reggio Emilia Uveitis Spondyloarthritis Interdisciplinary Approach (REUSIA) algorithm, proposed in this study and under validation, may represent a new tool to guide the selection of appropriate candidates for referral to rheumatology and to allow earlier diagnosis.

**Figure 1:**
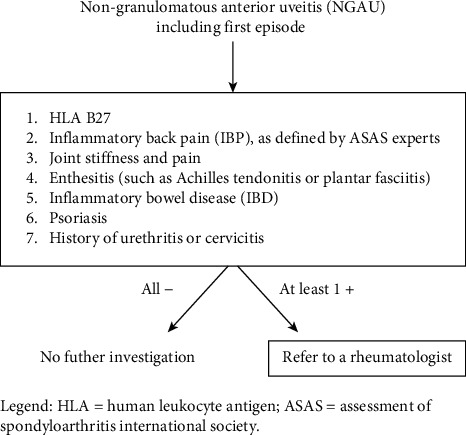
Reggio Emilia Uveitis Spondyloarthritis Interdisciplinary Approach (REUSIA) algorithm. Legend: HLA: human leukocyte antigen; ASAS: Assessment of SpondyloArthritis international Society.

Based on our experience, we recommend that the ophthalmologist, after a correct diagnosis of NGAU, refer all patients with HLA-B27 positivity and/or IPB and/or joint stiffness and pain and/or enthesitis (such as Achilles tendonitis or plantar fasciitis) and/or a history of psoriasis and/or IBD to rheumatology (REUSIA algorithm, Figure 1).

The study was limited by the small number of patients and the predominantly female sex of the patients. Prospective investigations to evaluate and validate this referral algorithm are needed.

## Figures and Tables

**Table 1:** Main characteristics of study population according to SpA diagnosis.

	Total (*n* = 99)		SpA- (*n* = 63)		SpA+ (*n* = 36)		*p* value
	Mean ± SD/median (IQR)	*N* (%)	Mean ± SD/median (IQR)	*N* (%)	Mean ± SD/median (IQR)	*N* (%)	
Age (yrs)	46 ± 13		48 ± 12		43 ± 13		0.05
Age < 45 yrs		47 (48)		25 (40)		22 (61)	0.04^∗^
Age at uveitis diagnosis (yrs)	42 ± 13		45 ± 12		37 ± 14		0.005^∗∗^
Uveitis diagnostic delay (mo)	3 (0-32)		5 (0-36)		1 (0-23)		0.28
Sex							
M		38 (38)		24 (38)		14 (38)	0.94
F		61 (62)		39 (62)		22 (62)	
BMI (kg/m^2^)	25 ± 5		25 ± 5		25 ± 6		0.88
Family history of SpA^†^		39 (39)		22 (35)		17 (47)	0.23
Current smoker		23 (23)		14 (22)		9 (25)	0.80
Diabetes mellitus		3 (3)		2 (3)		1 (3)	0.91
Dyslipidemia		25 (25)		16 (25)		9 (25)	0.75
Hypertension		13 (13)		9 (14)		4 (11)	0.65
HLA-B27 +		60 (60)		30 (48)		30 (83)	<0.001^∗∗∗^
Axial SpA		22 (22)		0 (0)		22 (61)	Nd
Peripheral SpA		14 (14)		0 (0)		14 (39)	Nd
IBP (ASAS criteria)		24 (24)		2 (3)		22 (61)	<0.001^∗∗∗^
Enthesitis		13 (13)		0 (0)		13 (36)	<0.001^∗∗∗^
Dactylitis		7 (7)		0 (0)		7 (19)	<0.001^∗∗∗^
IBD		5 (5)		5 (8)		0 (0)	0.22
Psoriasis		10 (10)		7 (11)		3 (8)	0.66
Urethritis/cervicitis		12 (12)		7 (11)		5 (14)	0.68
Peripheral arthritis		19 (19)		0 (0)		19 (53)	<0.0001^∗∗∗^
Ophthalmic evaluation:							
Uveitis							
Acute		29 (29)		16 (25)		13 (36)	0.26
Recurrent		70 (71)		47 (75)		23 (64)	
Bilateral uveitis		40 (40)		27 (43)		13 (36)	0.51
Hypopyon		2 (2)		0 (0)		2 (6)	0.06
Synechiae		29 (29)		21 (33)		8 (22)	0.24
Cataract		19 (19)		12 (19)		7 (19)	0.96

Legend: SD: standard deviation; IQR: interquartile range; SpA: spondyloarthritis; F: female; M: male; BMI: body max index; HLA: human leukocyte antigen; IBP: inflammatory back pain; ASAS: Assessment of SpondyloArthritis international Society; IBD: inflammatory bowel disease. ^†^Family history of AS, psoriasis, reactive arthritis, uveitis, or IBD in a first-degree relative (father, mother, sisters, brothers, and children) or second-degree relative (maternal and paternal grandparents, aunts, uncles, nieces, and nephews).

**Table 2:** Main characteristics of study population according to male sex.

	Total males (*n* = 38)		SpA- (*n* = 24)		SpA+ (*n* = 14)		*p* value
	Mean ± SD/median (IQR)	*N* (%)	Mean ± SD/median (IQR)	*N* (%)	Mean ± SD/median (IQR)	*N* (%)	
Age (yrs)	48 ± 12		51 ± 10		44 ± 14		0.08
Age < 45 yrs		25 (66)		19 (79)		6 (43)	0.02^∗^
Age at uveitis diagnosis (yrs)	45 ± 11						0.02^∗^
Uveitis diagnostic delay (mo)	5.5 (0-35)		5.5 (0-31)		9.5 (0-50)		0.94
BMI (kg/m^2^)	26 ± 4		26 ± 4		27 ± 5		0.81
Family history of SpA^†^		11 (29)		7 (29)		4 (29)	0.97
Current smoker		11 (29)		8 (35)		3 (23)	0.75
Diabetes mellitus		1 (3)		1 (4)		0 (0)	0.44
Dyslipidemia		7 (18)		5 (21)		2 (14)	0.72
Hypertension		5 (13)		4 (17)		1 (7)	0.40
HLA-B27 +		21 (55)		9 (38)		12 (86)	0.004^∗∗^
Axial SpA		9 (64)		0 (0)		9 (64)	Nd
Peripheral SpA		5 (13)		0 (0)		5 (36)	Nd
IBP (ASAS criteria)		10 (26)		1 (4)		9 (64)	<0.001^∗∗∗^
Enthesitis		5 (13)		0 (0)		5 (36)	0.002^∗∗^
Dactylitis		3 (8)		0 (0)		3 (21)	0.02^∗^
IBD		2 (5)		2 (8)		0 (0)	0.54
Psoriasis		4 (11)		2 (8)		2 (14)	0.56
Urethritis/cervicitis		5 (13)		3 (13)		2 (14)	0.87
Peripheral arthritis		5 (13)		0 (0)		5 (36)	0.002^∗∗^
Ophthalmic evaluation:							
Uveitis							
Acute		11 (29)		8 (35)		3 (21)	0.44
Recurrent		27 (71)		16 (67)		11 (79)	
Bilateral uveitis		17 (45)		10 (42)		7 (50)	0.62
Hypopyon		0 (0)		0 (0)		0 (0)	Nd
Synechiae		10 (26)		7 (29)		3 (21)	0.60
Cataract		7 (18)		5 (21)		2 (14)	0.61

Legend: BMI: body max index; SpA: spondyloarthritis; HLA: human leukocyte antigen; IBP: inflammatory back pain; ASAS: Assessment of SpondyloArthritis international Society; IBD: inflammatory bowel disease. ^†^Family history of AS, psoriasis, reactive arthritis, uveitis, or IBD in a first-degree relative (father, mother, sisters, brothers, and children) or second-degree relative (maternal and paternal grandparents, aunts, uncles, nieces, and nephews).

**Table 3:** Main characteristics of study population according to female sex.

	Total females (*n* = 61)		SpA- (*n* = 39)		SpA+ (*n* = 22)		*p* value
	Mean ± SD/median (IQR)	*N* (%)	Mean ± SD/median (IQR)	*N* (%)	Mean ± SD/median (IQR)	*N* (%)	
Age (yrs)	44 ± 13		46 ± 12		42 ± 13		0.27
Age < 45 yrs		27 (44)		19 (49)		8 (36)	0.35
Age at uveitis diagnosis (yrs)	40 ± 14		43 ± 13		35 ± 15		0.04^∗^
Uveitis diagnostic delay (mo)	2 (0-24)		3 (0-40)		1 (0-8)		0.18
BMI (kg/m^2^)	24 ± 5		24 ± 5		24 ± 6		0.99
Family history of SpA^†^		28 (46)		15 (39)		13 (59)	0.12
Current smoker		12 (20)		6 (15)		6 (27)	0.41
Diabetes mellitus		2 (3)		1 (3)		1 (5)	0.68
Dyslipidemia		18 (30)		11 (28)		7 (32)	0.77
Hypertension		8 (13)		5 (13)		3 (14)	0.93
HLA-B27 +		39 (64)		21 (54)		18 (82)	0.03∗
Axial SpA		13 (21)		0 (0)		13 (59)	Nd
Peripheral SpA		9 (41)		0 (0)		9 (41)	Nd
IBP (ASAS criteria)		14 (23)		1 (3)		13 (59)	<0.001^∗∗∗^
Enthesitis		8 (13)		0 (0)		8 (36)	<0.001^∗∗∗^
Dactylitis		4 (7)		0 (0)		4 (18)	0.006^∗∗^
IBD		3 (5)		3 (8)		0 (0)	0.41
Psoriasis		6 (10)		5 (13)		1 (5)	0.30
Urethritis/cervicitis		7 (12)		4 (10)		3 (14)	0.69
Peripheral arthritis		14 (23)		0 (0)		14 (64)	<0.001^∗∗∗^
Ophthalmic evaluation:							
Uveitis							
Acute		18 (30)		8 (21)		10 (45)	0.04^∗^
Recurrent		43 (70)		31 (79)		12 (55)	
Bilateral uveitis		23 (38)		17 (44)		6 (27)	0.21
Hypopyon		2 (3)		0 (0)		2 (9)	0.06
Synechiae		19 (31)		14 (36)		5 (23)	0.29
Cataract		12 (20)		7 (18)		5 (23)	0.65

Legend: SD: standard deviation; IQR: interquartile range; BMI: body max index; SpA: spondyloarthritis; HLA: human leukocyte antigen; IBP: inflammatory back pain; ASAS: Assessment of SpondyloArthritis international Society; IBD: inflammatory bowel disease. ^†^Family history of AS, psoriasis, reactive arthritis, uveitis, or IBD in a first-degree relative (father, mother, sisters, brothers, and children) or second-degree relative (maternal and paternal grandparents, aunts, uncles, nieces, and nephews).

**Table 4:** Main characteristics of study population according to SpA classification.

	Axial SpA (*n* = 22)		Peripheral SpA (*n* = 14)		*p* value
	Mean ± SD/median (IQR)	*N* (%)	Mean ± SD/median (IQR)	*N* (%)	
Age (yrs)	45 ± 10		40 ± 16		0.13
Age < 45 yrs		10 (46)		4 (29)	0.31
Age at uveitis diagnosis (yrs)	39 ± 9		34 ± 20		0.14
Uveitis diagnostic delay (mo)	1 (0-34)		2 (0-8)		0.61
BMI (kg/m^2^)	25 ± 6		26 ± 5		0.58
Family history of SpA^†^		11 (50)		6 (43)	0.68
Current smoker		3 (14)		6 (43)	0.048^∗^
Sex					
M		9 (41)		5 (36)	0.76
F		13 (59)		9 (64)	
Diabetes mellitus		0 (0)		1 (7)	0.20
Dyslipidemia		4 (18)		5 (36)	0.24
Hypertension		1 (4)		3 (21)	0.12
HLA-B27		19 (86)		11 (79)	0.54
IBP (ASAS criteria)		22 (100)		0 (0)	<0.001^∗∗∗^
Enthesitis		8 (36)		5 (36)	0.97
Dactylitis		5 (23)		2 (14)	0.53
IBD		0 (0)		0 (0)	Nd
Psoriasis		1 (4)		2 (14)	0.30
Urethritis/cervicitis		2 (9)		3 (21)	0.30
Peripheral arthritis		6 (27)		13 (93)	<0.001^∗∗∗^
Ophthalmic evaluation:					
Uveitis					
Acute		8 (36)		5 (36)	0.97
Recurrent		14 (64)		9 (64)	
Uveitis laterality		9 (41)		4 (29)	0.45
Hypopyon		2 (9)		0 (0)	0.25
Synechiae		5 (23)		3 (21)	0.93
Cataract		4 (18)		3 (21)	0.81

Legend: SpA: spondyloarthritis; SD: standard deviation; IQR: interquartile range; BMI: body max index; M: male; F: female; HLA: human leukocyte antigen; IBP: inflammatory back pain; ASAS: Assessment of SpondyloArthritis international Society; IBD: inflammatory bowel disease. ^†^Family history of AS, psoriasis, reactive arthritis, uveitis, or IBD in a first-degree relative (father, mother, sisters, brothers, and children) or second-degree relative (maternal and paternal grandparents, aunts, uncles, nieces, and nephews).

**Table 5:** Main characteristics of study population according to HLA-B27 status.

	HLA-B27- (*n* = 39)		HLA-B27+ (*n* = 60)		*p* value
	Mean ± SD/median (IQR)	*N* (%)	Mean ± SD/median (IQR)	*N* (%)	
Age (yrs)	47 ± 11		45 ± 13		0.41
Age < 45 yrs		24 (62)		28 (47)	0.15
Age at uveitis diagnosis (yrs)	45 ± 12		40 ± 14		0.049^∗^
Uveitis diagnostic delay (mo)	2 (0-24)		3.5 (0-33)		0.41
Sex					
M		17 (44)		21 (35)	0.39
F		22 (56)		39 (65)	
BMI (kg/m^2^)	26 ± 5		24.5 ± 5		0.08
Family history of SpA^†^		14 (36)		25 (42)	0.57
Current smoker		14 (36)		9 (15)	0.02^∗^
Diabetes mellitus		1 (3)		2 (3)	0.83
Dyslipidemia		11 (28)		14 (23)	0.43
Hypertension		7 (18)		6 (10)	0.25
Axial SpA		3 (8)		19 (32)	0.005^∗∗^
Peripheral SpA		3 (8)		11 (18)	0.14
SpA		6 (15)		30 (50)	<0.0001^∗∗∗^
IBP (ASAS criteria)		5 (13)		19 (32)	0.01^∗^
Enthesitis		3 (8)		10 (17)	0.20
Dactylitis		1 (3)		6 (10)	0.16
IBD		2 (5)		3 (5)	0.93
Psoriasis		4 (10)		6 (10)	0.97
Urethritis/cervicitis		5 (13)		7 (12)	0.86
Peripheral arthritis		4 (10)		15 (25)	0.07
Ophthalmic evaluation:					
Uveitis					
Acute		13 (33)		16 (27)	0.48
Recurrent		26 (67)		44 (73)	
Uveitis laterality		18 (46)		22 (37)	0.35
Hypopyon		0 (0)		2 (3)	0.25
Synechiae		17 (44)		12 (20)	0.01^∗^
Cataract		9 (23)		10 (17)	0.43

Legend: HLA: human leukocyte antigen; SD: standard deviation; IQR: interquartile range; M: male; F: female; BMI: body max index; SpA: spondyloarthritis; IBP: inflammatory back pain; ASAS: Assessment of SpondyloArthritis international Society; IBD: inflammatory bowel disease. ^†^Family history of AS, psoriasis, reactive arthritis, uveitis, or IBD in a first-degree relative (father, mother, sisters, brothers, and children) or second-degree relative (maternal and paternal grandparents, aunts, uncles, nieces, and nephews).

## Data Availability

Data are available on request.
